# A vehicle to vehicle relay-based task offloading scheme in Vehicular Communication Networks

**DOI:** 10.7717/peerj-cs.486

**Published:** 2021-04-13

**Authors:** Salman Raza, Muhammad Ayzed Mirza, Shahbaz Ahmad, Muhammad Asif, Muhammad Babar Rasheed, Yazeed Ghadi

**Affiliations:** 1 State Key Laboratory of Networking and Switching Technology, Beijing University of Posts and Telecommunications, Beijing, China; 2 School of Electronic Engineering, Beijing University of Posts and Telecommunications, Beijing, China; 3 Department of Computer Science, National Textile University, Faisalabad, Pakistan; 4 Computer Engineering Department, University of Alcalá, Madrid, Spain; 5 Department of Electronics and Electrical Systems, The University of Lahore, Lahore, Pakistan; 6 Department of Software engineering/Computer Science, Al Ain University of Science and Technology, Abu Dhabi, UAE

**Keywords:** Vehicular edge computing, Mobile edge computing, Vehicular cloud computing, Vehicular adhoc network, Task offloading

## Abstract

Vehicular edge computing (VEC) is a potential field that distributes computational tasks between VEC servers and local vehicular terminals, hence improve vehicular services. At present, vehicles’ intelligence and capabilities are rapidly improving, which will likely support many new and exciting applications. The network resources are well-utilized by exploiting neighboring vehicles’ available resources while mitigating the VEC server’s heavy burden. However, due to the vehicles’ mobility, network topology, and the available computing resources change rapidly, which are difficult to predict. To tackle this problem, we investigate the task offloading schemes by utilizing vehicle to vehicle and vehicle to infrastructure communication modes and exploiting the vehicle’s under-utilized computation and communication resources, and taking the cost and time consumption into account. We present a promising relay task-offloading scheme in vehicular edge computing (RVEC). According to this scheme, the tasks are offloaded in a vehicle to vehicle relay for computation while being transmitted to VEC servers. Numerical results illustrate that the RVEC scheme substantially enhances the network’s overall offloading cost.

## Introduction

The progress in the Internet of Things and wireless technologies put forward us prevalent smart devices like smart vehicles, which can execute numerous powerful and innovative applications (Raza et al., 2019). These applications include infotainment, automatic driving, and traffic cognition (Zhou et al., 2017). However, as resources are ever-increasing and performance requirements are more robust. Therefore, it is difficult for resource-constrained vehicles to support such intensive computing applications (Fuqiang & Lianhai, 2020).

Mobile cloud computing is introduced to cope with extensive computation requirements and greatly enhances computation performance and resource utilization. However, It also has its limitations due to the latency constraints while communicating with backbone networks. Since cloud servers are at a great distance from moving vehicles, this would lead to offloading effectiveness. Edge computing is presented as a way out, whereby it brings the services of the cloud to the edge of the network (Feng et al., 2017; Anavar et al., 2018) and facilitates computational offloading in the vicinity of the mobile vehicular networks (Shakarami et al., 2020). In VEC networks, each computation task has its own set of resource requirements (computational resources for executing the task and communication resources for its transmission) as VEC servers operate near to radio access networks and transfer task files with the help of associated roadside units (RSUs) (Zhou et al., 2020). Therefore, their operational areas may be confined by RSUs radio coverage. Vehicles are highly mobile, and they cross various RSUs and VEC servers on their way, so the computation tasks might be offloaded to any of the VEC servers, which they can access (Liu et al., 2020).

Due to the advancements in the internet of vehicles, there are more smart vehicles. These vehicles are armed with the computation unit, multi-communication technology, sensor platform, and human-machine interaction devices. With such progress in technology, it is more feasible for these smart vehicles to provide smart traffic applications, e.g., parking decisions, monitoring road traffic, and automatic management. Moreover, different multimedia onboard applications exist for both passengers and drivers (Boukerche & Soto, 2020). Such applications need high-grade computation. They have delay limitations, particularly the ones that involve real-time interaction and video processing like Image assisted navigation, immersive applications, and natural language processing (Shakarami et al., 2020).

**Figure 1:**
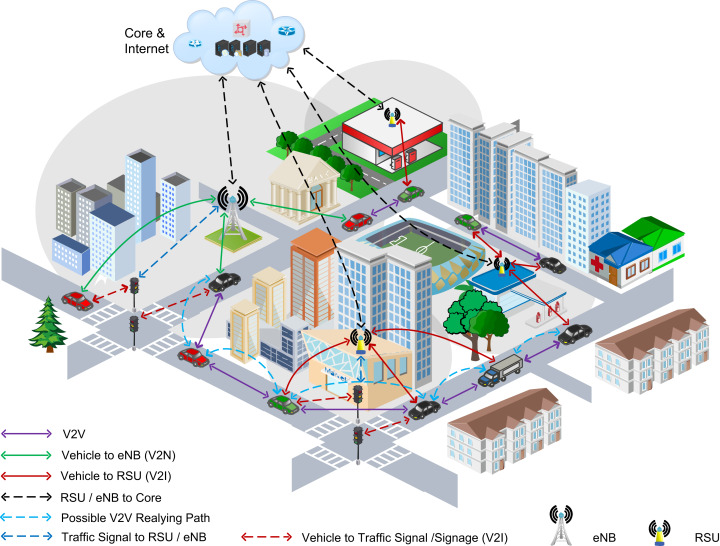
Depiction of Vehicle-to-Vehicle (V2V) relaying in task offloading in an urban vehicular edge computing environment.

The applications having too stringent computation requirements in computation present many constraints to the vehicular terminals, specifically their computational resources. Since vehicles have low computation capacity and limited storage compared to VEC servers (Raza et al., 2020). To address the ever-growing computation requirement of such applications, task offloading to VEC servers via the vehicular cloud is an exciting idea. However, exploiting the underutilized computational resources in a VEC environment is also useful for reducing VEC congestion and overall system cost (Raza et al., 2019). Also, vehicles often meet together for various purposes, such as passing through toll stations, waiting for traffic lights, or attracted to a particular area specifically in an urban environment. For instance, as we can observe in Fig. 1, the vehicles travel together due to traffic lights and can utilize resources for a short time. These vehicles might have unused resources for computing the tasks (Li et al., 2018). Accordingly, vehicles can offload the task to those relay vehicles and enable the resource constraint vehicle and relaying vehicles to cooperatively complete the task to minimize the task completion time (Guo et al., 2017).

### Motivation

VEC servers provide reduced transmission cost and quick response to the computation offloading service due to proximity. Nevertheless, compared to the conventional cloud, the VEC server still faces resource limitations. VEC servers have a tight time duration to perform computation tasks, particularly for the VEC servers, which are situated in highly dense road segments and experience various computation demands. Also, vehicles can communicate in a relay via V2V multi-hop connections as a considerable number of vehicles present on the road. Therefore, exploiting the benefit of V2V communication and using underutilized computational resources leads in terms of load balancing and latency reduction in vehicular networks. Moreover, unlike that, some operators provide RSU access services, V2V communication is self-organized by moving vehicles, and its cost is lesser than Vehicle-to-Infrastructure (V2I) communication.

### Contribution

Motivated by the facts mentioned above, we present a relay task/computation offloading scheme in vehicular edge computing (VEC). Figure 1 illustrates the task offloading through our proposed scheme. According to the scheme, the vehicles forward the input task files in their moving direction to the VEC servers through V2V multi-hop. The relay vehicle checks whether it has computational resources available to compute the task while transferring the task file. If it has sufficient resources, it computes the task and sends the desired vehicle’s output file.

They are otherwise based on the precise file transmission prediction and period spent by the vehicle while on the road. The other vehicle may enter the transmission range of RSU_n the moment its task is completed. The computation result is transferred straightly from RSU_n to the other vehicle via V2I transmission without multi-hop backhaul. Hence, using both computation and communication resources of the vehicle can reduce the overall system time and cost for the task offloading and network congestion. However, the duration of transmission delay of V2V and V2I modes depends on the state of wireless channels, the size of the input file, and the density of the road since beacons are the packets sent periodically in a broadcast by vehicles to share its information like type, speed, direction computation capacity, and channel state (Raza et al., 2020).

This paper put forward a promising relay task offloading scheme in vehicular edge computing (RVEC). In this work, both the heterogeneous demands of computation and communication tasks are examined by taking into account the vehicle dynamics. This scheme enhances transmission efficacy while meeting the computation tasks’ delay constraint. Moreover, RVEC reduces the overall cost of the offloading process. The significant contributions of this article are summarized as follows:

- This paper aims to minimize the overall offloading cost, including the computation and communication cost, while meeting the VEC environment’s delay constraints to ensure vehicular user quality of experience (QoE).
- We proposed an RVEC scheme, which allows vehicles to transfer the task to their neighboring vehicle in their moving direction to the VEC servers via V2V multi-hop. The V2V computation is done in the neighboring vehicle case with sufficient resources, and the output returns to the vehicle in a relay fashion. This is conditional on the maximum delay tolerance and the vehicle’s stay time. This scheme the overall offloading latency and cost and network congestion.
- A distributed RVEC algorithm is designed for the proposed scheme. We evaluate the impact of various parameters on the RVEC algorithm by comparing it with different approaches. The results confirm that RVEC outperforms compared to the existing solutions to minimize offloading cost, i.e., computation latency and communication resource utilization.

The rest of this article is structured as follows. We review the related work in “Related Work”. The system model is described in “System Model”. In “Relay Task Offloading Scheme in VEC”, our proposed RVEC scheme is discussed, comprised of computation mode and communication mode. The distributed RVEC algorithm is presented in “The Distributed RVEC Algorithm”. We demonstrate numerical results in “Numerical Results” and conclude the paper in “Conclusion”.

## Related work

Recently, plenty of research has been done on the vehicular networks to use the underutilized vehicular resources. In Yu et al. (2015), the authors provided a model for the coalition game to manage vehicular resources and share them with various cloud service providers. The soft data fusion and cognitive radio in vehicular networks are used and developed a traffic offloading mechanism in a distributed environment for cognitive vehicular cloud networks in Cordeschi, Amendola & Baccarelli (2015). Integrating vehicle cloud and central cloud, in Zhang, Zhang & Du (2015), and offloading strategy was proposed to find unused resources and perform task mitigation. Furthermore, the authors prepared an architecture for vehicular fog computing that uses a corporation of near-user edge devices and vehicles to accomplish computation and communication. These studies are primarily concerned with utilizing the unused vehicular resources and did not address the VEC server’s overload.

The most encouraging technical method to enhance cloud computing efficiency, Mobile edge computing (MEC), has gathered the researchers’ substantial focus lately. Recent works on MEC aims at minimizing latency by offloading computation-intensive and latency-sensitive tasks to the neighboring RSUs or base stations for executing it remotely. In Mao, Zhang & Letaief (2016), while considering the offloading decision, a delay-optimal computation offloading algorithm was presented. The authors in Liu et al. (2016) presented a power-constrained delay minimization problem and formulated it as a one-dimensional search algorithm. To study MEC (Chen et al., 2016) analyzed the problem of multiuser MEC computation offloading into a multichannel wireless environment and considered a distributed game-theoretic offloading approach. In Nunna (2015) studied the collaborate MEC with fifth-generation to offer context-aware communication in real-time. Compared with a mobile edge cloud concerning the application execution time in Drolia et al. (2013), the core cloud performance is compared. In Cau et al. (2016), the MEC servers’ mechanism was developed to utilize cloud-based packets virtualization fully. In Chen et al. (2015), the authors developed an algorithm for offloading decision making for the computation-extensive tasks as a multiuser game and attained a Nash equilibrium. However, some prior studies analyzed to optimize the task offloading decisions based on VEC servers or vehicles by using their respected resources without simultaneously optimizing them.

A vehicular cloud network can manage massive computation-extensive tasks flexibly and in a virtual manner (Huang et al., 2016; Xia et al., 2015). The work in Zheng et al. (2015) presents a semi-Markov-based decision-making scheme for maximizing the expected reward calculation for computation resource allocation of a vehicular cloud computing network. The authors in Yu et al. (2015) presented a coalition game for resource management among cloud service providers, in which the resources of the cloud-enabled vehicular network are utilized effectively.

However, several studies emphasize vehicular cloud networks and MEC technologies; in contemporary literature, few papers provide MEC research with vehicular cloud networks. Moreover, the effect of several vehicular communication schemes on task offloading performance and vehicle mobility has been overlooked. However, in Zhang et al. (2017), the authors proposed a predictive combination-mode offloading scheme (PCMS), pinpointing the vehicles’ transmission channel and reducing the offloading cost. Contrary to these studies, we study the offloading mechanism in VEC and propose a relay task offloading scheme by fully utilizing the computation and communication resources of vehicles to enhance the QoE and reduce the overall cost network.

## System model

N $\rho_{i}$ c d $t_{\max}$ $t_{i,j,k}$ $R_{i,j,k}$ We consider a one-way road that has a continuous flow of traffic. The RSUs are located along the road. M is considered to be the distance between two RSUs where each RSU provides wireless access to the vehicles that are within its vicinity. The communication range is represented as M/2, and the road can be segregated into various length M segments. Under the V2I communication mode, the vehicles moving along a road can only approach the RSUs located along the given road segment.

The RSUs connect each other via an optical-fiber backbone, and a central controller is also installed that controls the RSUs (Zhang et al., 2016). Each RSU has an attached VEC server facilitating vehicles for roadside services and computation resource sharing but in a limited capacity. For various applications, computation’s input data size is much larger than the output, e.g., speech recognition (Chen et al., 2015). The task-input file may not be transferred among the RSUs to improve the transmission of wireless backhauls. For that reason, each VEC server only performs the computation for the RSU with which it is associated. Nevertheless, as the output data is smaller than input, it can be transferred from one RSU to another through wireless backhaul.

Every Vehicle runs with uniform speed along the road, facilitated by vehicular communication protocols like dedicated short-range communication standards (Kenney, 2011). The Poisson distribution is followed by the vehicles on the road (Kenney, 2011). The density of traffic concerning the number of vehicles per unit distance is λ. Every Vehicle has an extensive computation task performed either locally by the vehicular terminal, in a V2V relay, or by the VEC servers attached with the RSUs. Table 1 enlists the key notations, while Fig. 1 depicts a scenario of a relay-based task offloading scheme in the vehicular edge networks. We represent the computation task by $T=\left\{c,d,t_{\max}\right\}$, where *c* represents the required computational resources to complete task *T*. The *c* can be measured as the required number of CPU cycles, and the *d* in the equation shows the input file size of the computation task along with some necessary information on the computation task, e.g., the programing instructions, shared variables, methods, or link libraries, whereas $t_{\max}$ is the maximum latency tolerance of the task.

**Table 1:** The notations.

Symbol	Explanation
*N*	Total number of vehicle types
*ρ_i_*	The proportion of type-i tasks associated with vehicles
*c*	Required CPU cycles for task completion
*d*	Computation size of the input file.
$t_{\max}$	The task maximum delay tolerance
$t_{i,j,k}$	The time of the type-i task through relay vehicles
$R_{i,j,k}$	The cost of the type-i task through relay vehicles

We classify the S types of the tasks and define the tasks as $T_{i}=\left\{c_{i},d_{i},t_{i,\max}\right\},i\in N$, to analyze the task characteristics on the design of offloading schemes, *N* represents a total number of vehicle types. According to the type of computation task, the vehicles are similarly categorized into N types. The type-i tasks associated vehicles from entire vehicles present on the road have a proportion $\rho_{i}$, where i∈N and $\sum_{i=1}^{N}\rho_{i}=1$. Table 1 enlists the key notations while Fig. 1 depicts a scenario of a relay-based task offloading scheme in the vehicular edge networks.

## Relay task offloading scheme in vec

The RVEC comprises computation and communication modes. We discuss each mode in detail, which are as follows. Display style equations should be numbered consecutively, using Arabic numbers in parentheses.

### Computation mode

The task computation mode selection depends on the processing time requirement and its impending cost. Each vehicle can choose to accomplish its computation task either locally on its vehicular terminal, on another vehicle in a relay, or a VEC server. The task-completion mode selection depends on the processing time requirement and the corresponding cost.

#### Onboard computing

Here, we assume that all vehicles have homogeneous resources for computation. *C*_0_ represents the local vehicle resources. The vehicles with type-i tasks are categorized as $T_{i}$ vehicles. A type-i vehicle wishes to complete its task $T_{i}$ locally through its onboard computing, the execution time can be denoted by $t_{i,obc}=C_{i}/C_{0}$, and the cost of onboard computing is $R_{i,obc}$.

By satisfying (1), type-i vehicles will offload their computation tasks to other vehicles in a relay or to the VEC servers to execute the tasks under delay constraints.

(1) $$t_{i,obc}>t_{i,\max}.$$

#### Relay computing

We denote the relay vehicle computation resources as $C_{r}$. In this case, if a type-i vehicle wishes to perform a computation task $T_{i}$ through offloading and there are sufficient resources available to accomplish this task. The task’s execution time is denoted by $t_{i,relay}=C_{i}/C_{r}$, and the relay cost is represented as $R_{i,relay}$.

Moreover, if (2) is satisfied, type-i vehicles forward the tasks to the other vehicles or the VEC servers to complete those tasks through delay constraints.

(2) $$t_{i,relay}>t_{i,\max}.$$

#### VEC computing

When portions of the vehicles decide to select VEC computing mode, they offload VEC servers’ tasks. Let $P_{i,j}$ represent the probability of the offloaded tasks by the type-i vehicles to the VEC servers connected to the RSUs j that are far from their present location (Zhang et al., 2017).

The integral part of the offloading process’s delay is the time consumption of file transmission from the vehicles to the VEC servers. Furthermore, to transmit data, the distant VEC server implies increasingly wireless transmission hops and greater time latency. We denote $J_{i,\max}$ as the highest possible hop to the VEC servers, which type-i vehicles may offload tasks under the task’s delay constraints. The arrival and the fulfillment of the computing tasks on a VEC server follow an M/M/1 queue. According to this queuing design, for every VEC server, computation has an exponential distribution with a mean service time 1/μ. The time required to complete a task is given by:

(3) $$t_{execute}=1/\left(\mu -\lambda \right),$$

which indicates both the waiting and the computing execution time in a queue. Here is the average task arriving rate at a VEC server and is defined as:

(4) $$=\overset{N}{\underset{i=1}{\sum }}\overset{J_{i},\max}{\underset{j=1}{\sum }}P_{i,j}\rho_{i}\lambda .$$

The operators always provide different services, i.e., maintenance of VEC servers, computation, offloading, etc., by charging some fees against those facilities. Where the computing cost for a type-i task on the VEC servers is defined as $R_{i,execute}$.

### Communication mode

We compare V2I transmission with our proposed relay transmission approach concerning time consumption and cost to validate our approach. This work’s communication scheme is used to generate a realistic vehicular environment to study the task offloading problem. Moreover,

we examine practical assumptions and measure the transmission rates for relay transmission and V2I transmission according to our previous work (Raza et al., 2020).

#### Offloading through direct transmission

We consider a scenario in which a type-i vehicle travels on a road segment m. The vehicle can directly reach the in-range RSU located on the road segment. We represent this RSU as RSU m. If the mode followed by the vehicle is direct V2I mode, then it will directly transfer the file to the RSU m. The process of file uploading is more manageable, less time-consuming, and more cost-efficient. However, each computation task on the VEC server requires execution time $t_{i,execute}$. Since the vehicle moves with high mobility, it might get out of the communication range of the RSU m before the execution time $t_{i,execute}$. Therefore, the output data requires to be transferred from the RSU m to the RSU at the place of the vehicle's arrival. The transmission among these RSUs travel via wireless backhauls.

Let the time delay and the cost for transferring the type-i task output through one road segment are expressed by $t_{i,backhaul}$ and $R_{i,backhaul}$, respectively. The overall time consumption of task completion via V2I mode is defined as:

(5) $$t_{i,m}=t_{i,up}+t_{i,execute}+x_{i}\;\Delta \;t_{i,backhaul}+t_{i,down},$$

where $x_{i}$ represents the number of road segments that a vehicle passes via during the time sum of $t_{i,execute}$, $t_{i,up}$ and $t_{i,down}$. The overall cost of completion of the task in V2I mode is calculated as follows:

(6) $$R_{i,m}=R_{i,up}+R_{i,execute}+x_{i}\;\Delta \;R_{i,backhaul}+R_{i,down}.$$

#### Offloading relay transmission

As the vehicles’ speed is high and tasks take more time in their execution, the vehicle gets out of the VEC server’s proximity that executes the task. A longer delay might incur additional cost and transfer process.

In such cases, when a vehicle offloads its task, it is first transmitted through the multi-hop V2V relay transmission. As a worst-case scenario, if no vehicles have enough computational resources in the relay, they keep on transferring the task to the next vehicle in a relay. Until the file is transferred to an RSU via V2I by the vehicle at the transmission relay’s destination hop. Therefore, the computation task is offloaded in advance of time to the VEC server in the vehicle’s headway compared to the vehicle’s current position. After the computation result/output reception, the VEC server saves the output to the RSU associated with it. Whenever the offloading vehicle enters into the communication range of RSU, it takes the output directly from the RSU.

Let $t_{i,v2v}$ represents the average time delay in transmitting type-i input task files via a one-hop V2V relay to the destination VEC. The overall time consumption of type-i task accomplishment to VEC offloading is given as follows:

(7) $$t_{i,j}=y_{j}\;\Delta \;t_{i,v2v}+t_{i,up}+t_{i,execute}+t_{i,down},$$

where j is represented as the hop count to the upload destination RSU ahead from the vehicle’s present location. We represent $y_{j}$ as the V2V relay hops needed to transfer the input task file to the j-hop away RSU. Additionally, the total cost of the type-i task to VEC offloading can be similarly given as:

(8) $$R_{i,j}=y_{j}\Delta R_{i,v2v}+R_{i,up}+R_{i,execute}+R_{i,down}.$$

Contrary to this, if any vehicle in the relay has sufficient computation resources available, it will compute the task file while moving on the road. After completing the task, the vehicle sends the output to the relay’s desired vehicle and earns some reward for both computation and transmission. This process can reduce the overall cost and time to execute the task. The time consumption of the type-i task through relay vehicles will be defined as:

(9) $$t_{i,j,k}=y_{j}\Delta t_{i,v2v}+t_{i,relay}+y_{k}\Delta t_{i,v2v}.$$

Likewise, the cost would be defined as:

(10) $$R_{i,j,k}=y_{j}\Delta R_{i,v2v}+R_{i,relay}+y_{k}\Delta R_{i,v2v}.$$

By comparing Eqs. (9) & (7) and (10) & (8), we can interpret that if a vehicle in a relay has sufficient resources, then the task would be computed by exploiting the underutilized vehicular resources since the vehicle gets its output before reaching its destination, RSU. Therefore, it is less time-consuming and more cost-efficient. On the other hand, with comparison to the Eqs. (9) & (5) and (10) & (6), it can be figured out that Eq. (5) uses computational network resources, while the time of uploading $t_{i,up}$, downloading $t_{i,down}$, and backhaul $t_{i,backhaul}$ will also be saved in Eq. (9). The cost factor will also work on the same principle as we neither use computational nor the network’s communication resources.

The total offloading cost for our scheme is defined as:

(11) $$R=min\left\{R_{i,m},R_{i,j,k}\right\}.$$

Thus, offloading cost minimization problem for the proposed relay task offloading scheme can be formulated as:

$$P1:\underset{\left(t_{i,j},t_{i,j,k}\right)}{min}R$$

(12) $$s.t.\;t_{i,obc},t_{i,j,k}\leq t_{i,\max},$$

(13) $$0\leq C_{i}\leq C_{0},C_{i}\leq C_{r},C_{i}\leq C_{VEC},\;\forall n\in N,$$

Here, (12) denotes that the computation time for local, relay, and VEC server's computation time should not exceed the maximum allowable delay tolerance. (13) Confirms that the allocated computing resource for the vehicle i would not exceed the comprehensive local resource, neighboring vehicle, and VEC server, respectively.

## The distributed rvec algorithm

Here, by taking advantage of the neighboring vehicles’ communication and computational resources, we design an effective algorithm identified as a distributed RVEC Algorithm. The main objective of the distributed RVEC Algorithm is to minimize the network’s offloading cost.

Algorithm 1 presents a complete description of the proposed task offloading scheme. This algorithm aims to fully utilize the vehicular resources to provide the ease to overburden VEC servers. In Algorithm 1, each vehicle has a task that is computed locally or offloaded to the neighboring vehicle for computation or the VEC server via the V2V relay or directly. The process remains conditional to the maximum delay tolerance. Here, Lines 3–5 are used to compute the task locally, and Lines 6–22 are used to offload the task. Line 8 indicates that the task is transmitted in V2V for maximum hop in a relaying fashion.

**Algorithm 1:** RVEC algorithm.

1	*J* = 0 for onboard computation, and *J* = 1 Choose to offload task.				
2	**while** *t*_*i*,max_ **do**				
3		**if** *J* = 0 **then**			
4			Local computation		
5		**end if**			
6		**if** *J* = 1 **then**			
7			Offloads the Task-file to the relay vehicle		
8			**while** *!P*_*i,j*_ **do**		
9				**if** *t*_*i,j,k*_ <= *t*_*i*,max_ **then**	
10					*Vehicle*_*i*_ will compute this Task-file
11					Send output via V2V Relay to desired Vehicle
12					*Vehicle*_*i*_ gets reward for providing its both transmission and computation resources
13					exit()
14				**else**	
15					*Vehicle*_*i*_ will transfer the Task-file to next vehicle
16					*Vehicle*_*i*_ gets reward for providing its transmission resources
17				**end if**	
18			**end while**		
19			Offload task file to VEC server		
20			VEC Compute the task file		
21			Send output to Vehicle through RSU		
22		**end if**			

Moreover, Line 9–13 represent that if the time in relay computation is less than that of maximum delay tolerance, then the vehicle having sufficient computation resources computes the task and provides the output to the initial vehicle in the relay and gets the reward for providing its communication and computational resources from the service provider. The reward is received in terms of, i.e., electronic cash, free provision of network resources in the future, etc. On the other hand, Line 14–17 illustrates that if the vehicle is unable to be computed, it transmits the task to the next vehicle in the relay and earns its reward for only providing communication resources. Line 19–21 indicates that if none of the relaying vehicles can compute. Then the task will be transferred to the VEC server for computation. After the desire computation, the VEC server transfers back the initial vehicle results via the corresponding RSU.

**Figure 2:**
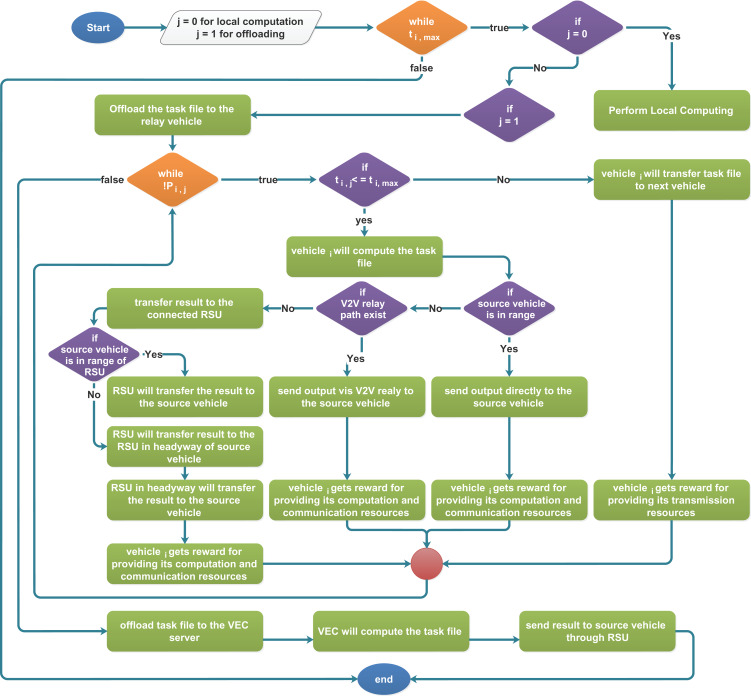
RVEC flowchart.

The flowchart of Algorithm 1 is represented in Fig. 2, which illustrates the algorithm more clearly. Also, it is worth noting that a vehicle recognizes its neighboring vehicles through the beacons since beacons are the packets sent periodically in a broadcast by vehicles to inform its type, speed, computation capacity, location, and channel state (Raza et al., 2020).

## Numerical results

We demonstrate the performance results of our RVEC scheme in this section. We take five RSUs positioned alongside a unidirectional urban road. The vehicles are moving at a speed of 120 Km/h, and the vehicle’s density on the road is considered as λ = 0.3. The vehicle’s computational tasks are categorized into four types with the probabilities {0.01,0.2,0.3,0.4}. We figure out each task type’s computation resource as {9,18,26,33} units, respectively, other simulation parameter values are shown in Table 2. Since the resource requirement is the vital factor influencing offloading performance.

**Table 2:** System parameters.

Parameter	Value
No. of RSUs	5
The transmission power of each Vehicle	24 dBm
Computation capacity of a VEC	[3.10] MIPS
Computation capacity of a vehicle	[1.3] MIPS
Wireless transmission speed	10 MB/s
Task input size	[2.5] MB

The proposed RVEC scheme is evaluated against the following benchmark schemes.

- V2I Direct scheme: In this scheme, the task is directly transmitted to the vehicle’s VEC server. Therefore, this scheme ignores utilizing the neighboring vehicles’ computation resources.
- PCMS Scheme: This scheme utilizes the vehicular communication resource while ignoring the vehicular computation resources. Thus, the task is only computed on VEC servers in a V2V relay manner.

Figure 2 analyses the total task offloading costs concerning vehicle density (λ) on the road. We make a comparison of our proposed RVEC scheme with the V2I direct and PCMS (Zhang et al., 2017). It can easily be seen that when the density of the vehicle (λ) is high, then the RVEC dramatically reduces the overall offloading cost. Nevertheless, the cost-effectiveness is not satisfactory while having low density (λ). Since the number of vehicles on the road is limited, the difference between all schemes’ offloading costs is minor. Besides, the burden of computation on every VEC server is low. A large percentage of the VEC servers’ tasks may be computed in the specific period while the vehicles are accessing their corresponding RSUs.

On the other hand, in the case where the (λ) has a high value, the moving vehicles may pass more RSUs during a long time of task execution on VEC servers. Due to communication and computation, the wireless backhaul cost among RSUs, the direct V2I offloading scheme’s overall costs rise fast as the λ grows. Moreover, in PCMS, part of the transmission is offloaded to the vehicular relay, which has a lesser cost than the wireless backhaul communication. However, the results reveal that the proposed scheme distinctly reduces computation and communication time and offloading system cost. Therefore, using the RVEC scheme, the overall offloading cost can effectively be saved.

**Figure 3:**
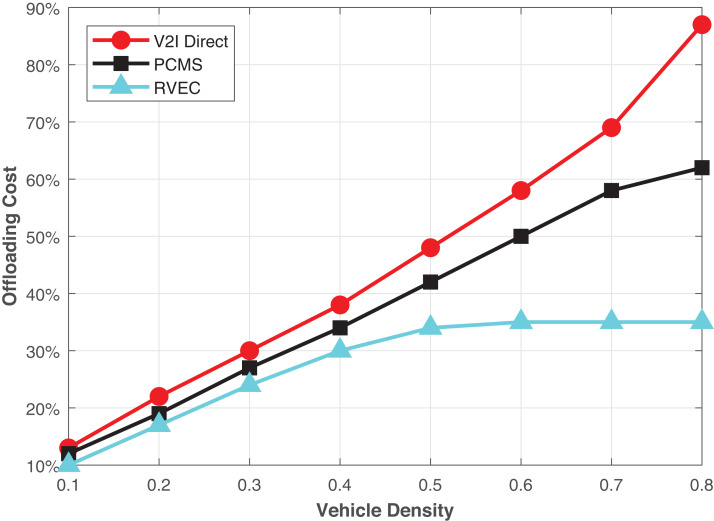
The offloading costs in terms of vehicles density.

Figure 3 presents the percentage of different types of tasks offloaded via RVEC transmission. We take a low priority task as type 1 to the highest priority task as type 4, respectively, in terms of the real-time response, i.e., safety application and non-safety application. The more critical processes required a prompt response; thus, they prefer to offload with the RVEC scheme. Moreover, it becomes more advantageous to increase vehicles’ density (λ). The VEC servers are at a heterogeneous level of computation load. When the vehicles take up V2I direct, the higher priority-type index tasks may take a longer time to complete their job. Thus, more RSUs may pass through as vehicles move at high speed. The extra cost of backhaul transmission will be induced to the system. On the other hand, if the vehicle chooses the PCMS scheme, the vehicle still consuming the computation resources of VEC and increases the burden. Moreover, those tasks that rapid response will not be treated until the vehicle reaches the communication area of the destination RSU.

**Figure 4:**
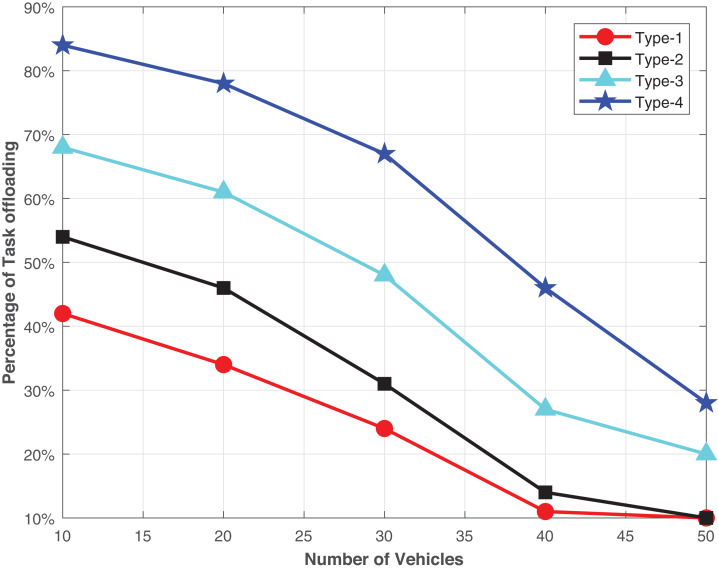
The offloading costs in terms of the relay task offloading in vehicular edge computing.

In Fig. 4, the offloading cost with different speeds of the vehicles is illustrated. It can be observed that the offloading cost increase as the speed of the vehicle increases. Due to strict transmission delay constraints, the number of relay vehicles decreases as the vehicle speed increases. Consequently, the vehicle is unlikely to match with a vehicle with adequate resources to perform computation, and the VEC server can only process the tasks. Therefore, the proposed scheme is more suitable for all conditions than the other benchmark schemes.

**Figure 5:**
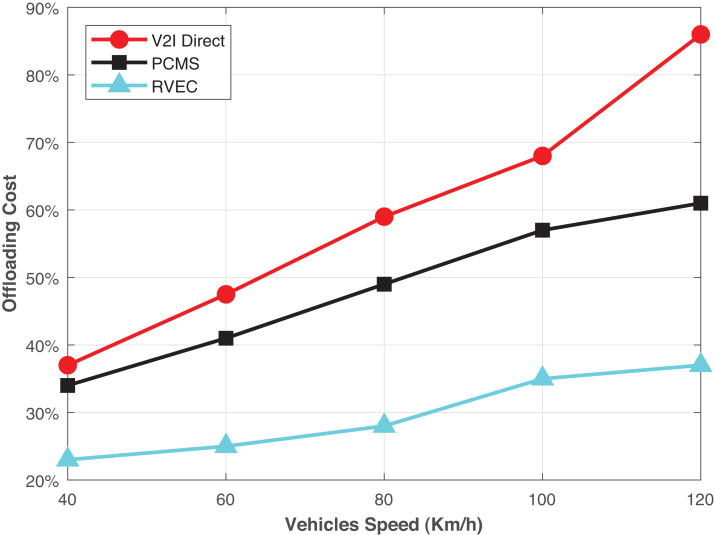
The offloading costs in terms of the vehicles’ speed.

In Fig. 5, the offloading cost in terms of different maximum delay tolerance $t_{\max}$ is presented. We observe that the higher the value of $t_{\max}$, the lower the offloading cost. We consider that more computation is done on V2V mode and need not pay for the communication and computation costs when they offload their tasks to the VEC servers. The performance of the RVEC scheme is much more reliable than other schemes. The results reveal that compared with the V2I Direct and PCMS algorithm, the RVEC algorithm can significantly minimize the offloading cost over the considered range of $t_{\max}$, by about 25% and 13%, respectively.

**Figure 6:**
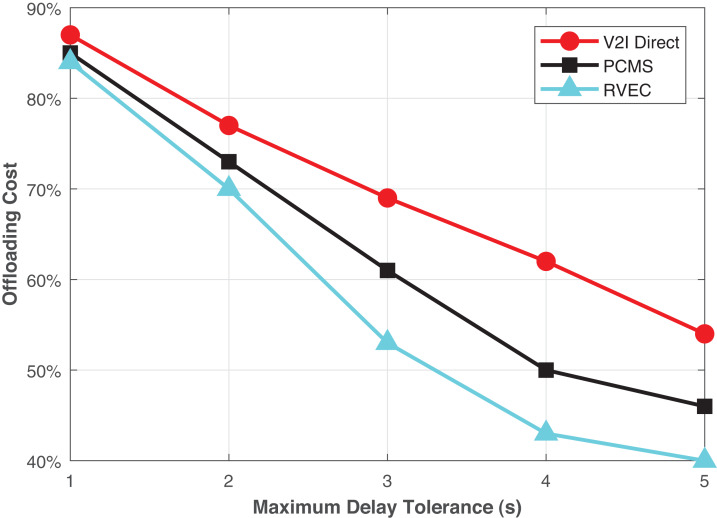
The offloading costs in terms of the maximum delay tolerance.

**Figure 7:**
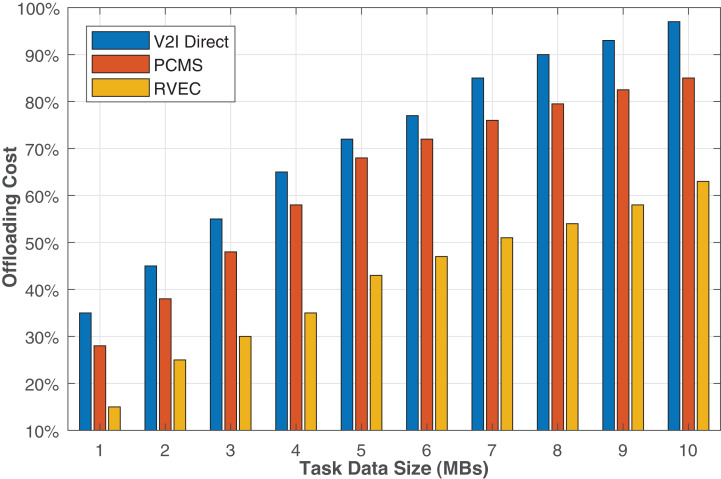
The offloading costs in terms of the task data size.

Figure 6 reveals that all algorithms’ offloading cost grows with the task data size. The larger the task’s size, the longer it will require accomplishing the task on the vehicle or a VEC server. Thus, it consumes more computation and communication resources. Moreover, each task offloaded to the other vehicles via V2V mode, or the VEC server takes more time offloading and takes more computation time. Therefore, an increase in task communication and computation time affects the system’s overall offloading cost. Consequently, the offloading cost rises exponentially. We can observe from Fig. 7 that the proposed algorithm remains cost-efficient from other schemes for all task sizes.

For the reasons mentioned above, using the RVEC scheme will relieve the communication and computing burden of the VEC servers, thus avoiding network congestion. The higher priority task is more likely to prefer the RVEC scheme to reduce the offloading cost.

## Conclusion

In contempt of the rapid response rate, the VEC servers usually confront the resource limitation compared to the conventional core-cloud with a comparatively large computational capacity. The VEC servers associated with RSUs located at the road segments have to serve a high density of vehicles, leading to increased latency and network congestion. To avoid this situation, we designed a task offloading strategy while taking advantage of a vehicle’s transmission and underutilized-computational resources. Furthermore, we investigated the time consumption and the offloading cost of different transmission modes and proposed a promising relay task offloading mechanism in VEC. Also, numerical results reveal that our scheme significantly reduces the offloading cost, i.e., computation latency and communication resource utilization, and outperforms other benchmark schemes. In our future work, we will study how to shift VEC server burdens to the vehicular cloud through incorporating a machine learning algorithm. By considering the dynamic vehicular nature, diverse application needs, and application stringent latency constraints in more practical and real trace-based complex road scenarios in our future work.

## Supplemental Information

10.7717/peerj-cs.486/supp-1 Supplemental Information 1 Simulation code used in the project.

## Additional Information and Declarations

### Data Availability

The following information was supplied regarding data availability: There was no data used in the project but a simulation was performed. The simulation code used is available in the Supplemental File.
